# Efficiency of ant-control agents in colony-level oral toxicity tests using *Tetramorium tsushimae* (Hymenoptera: Formicidae) for post-establishment control of the red imported fire ant, *Solenopsis invicta* (Hymenoptera: Formicidae)

**DOI:** 10.1007/s13355-022-00800-x

**Published:** 2022-10-09

**Authors:** Hironori Sakamoto, Koichi Goka

**Affiliations:** Biodiversity Division, National Institute for Environmental Studies, Onogawa 16-2, Tsukuba, Ibaraki 305-0053 Japan

**Keywords:** Invasive alien species, Fire ant, Chemical control, Fipronil, *Solenopsis*, Insect growth regulator

## Abstract

The red imported fire ant *Solenopsis invicta* Buren (Hymenoptera: Formicidae) causes serious damage worldwide as an invasive alien species. The species has expanded its range to the Pacific Rim since 2000s and Japan has faced its multiple introductions since 2017. While colony-level control methods are urgently needed, testing living colonies of the unestablished species is challenging especially due to various restrictions under the COVID-19 pandemic. Here, we proposed alternative long-term toxicity assays using artificial colonies of *Tetramorium tsushimae* Emery (Hymenoptera: Formicidae), a Japanese native species belonging to the same subfamily (Myrmicinae) as *S. invicta*. We conducted an acute toxicity test to determine if *T. tsushimae* is a suitable substitute for *S. invicta* using fipronil and found the LD_50_ value in *T. tsushimae* was close to that in *S. invicta*. Then, we conducted the long-term toxicity test with fipronil and two insect growth regulators (pyriproxyfen and etoxazole) using artificial colonies of *T. tsushimae*. All workers and larvae in the fipronil-treated colonies died within 3 days of treatment initiation. Emergence of new workers was observed after 18 days in the etoxazole-treated and control colonies, but not in the pyriproxyfen-treated colonies. We concluded that fipronil was the most promising insecticide for post-establishment control, and pyriproxyfen was effective as a toxic-bait agent for colony-level control.

**Supplementary Information:**

The online version contains supplementary material available at 10.1007/s13355-022-00800-x.

## Introduction

The negative impacts of invasive alien species on native ecosystems and biodiversity have been extensively documented in recent years (Bellard et al. 2016; Blackburn et al. 2019; Clavero and García-Berthou 2005). In particular, invasive alien ants (Hymenoptera: Formicidae) have spread widely in their invasion areas, causing serious damage not only to native ecosystems, but also to human life (Fournier et al. 2019; Holway et al. 2002; Suarez et al. 2010) . Invasion by invasive alien ants as a result of unintentional introduction associated with human activities has become a global issue (Bertelsmeier et al. 2017; Fournier et al. 2019; Holway et al. 2002; Suarez et al. 2010). Invasive alien ants have had diverse origins and times of invasion in Asia (Xu et al. 2022). For example, the tropical fire ant, *Solenopsis geminata* (Fabricius) (Hymenoptera: Formicidae), native to Central America and the southern US, globally spread to Asia in the sixteenth century (Gotzek et al. 2015). From South America, Argentine ant, *Linepithema humile* (Mayr) (Hymenoptera: Formicidae), came to Japan in the 1990s (Sugiyama 2000), whereas the browsing ant, *Lepisiota frauenfeldi* (Mayr) (Hymenoptera: Formicidae), arrived in Japan from southern Europe in the 2010s Ministry of the Environment The red imported fire ant, *Solenopsis invicta* Buren (Hymenoptera: Formicidae), is probably the invasive alien ant of most serious concern in Asia at present (Sakamoto and Goka 2021; Wetterer 2013; Wylie et al. 2020). The species originated in South America and was introduced to the USA in the 1930s (Tschinkel 2006; Wetterer 2013). In the USA, *S. invicta* causes a total of USD 6 billion in damage every year, including harm to human health, agriculture, and electrical equipment (Drees and Lard 2006; Gutrich et al. 2007). Its invasion of Asia and the Pacific Rim began with Australia in 2001 and continued with New Zealand in 2004 and Taiwan and China in 2005. It has become established in all of these countries but New Zealand, where early eradication programs were successful (Wylie et al. 2020). Furthermore, there have been reports of unintentional introductions of *S. invicta* into South Korea and Japan since 2017 (Lyu and Lee 2017; Sakamoto and Goka 2021; Ujiyama and Tsuji 2018). Since 2019, large colonies of *S. invicta* have been discovered at major international ports (Xu et al. 2022), including ports in Tokyo (Ministry of the Environment 2020a), Nagoya (Ministry of the Environment 2020b), and Osaka (Ministry of the Environment 2021a). There is an urgent need to institute some sort of control measure that will eradicate colonies that have already become established.

The most proven method to date of post-establishment control of *S. invicta* is chemical control via synthetic insecticides (Hoffmann et al. 2016; Sakamoto and Goka 2021). Toxic baits with slow-acting insecticides are considered to be effective, but selection of the most effective insecticides is key to successful control (Hoffmann et al. 2016). Although many insecticides effective against hymenopteran insects have been developed in recent years, few efficacy comparisons have been conducted using uniform toxicity assays. In our previous study, we conducted an acute toxicity assay in workers of *S. invicta* to compare the efficacies of a phenylpyrazole (fipronil), neonicotinoids (thiamethoxam and imidacloprid), an oxadiazine (indoxacarb), and an amidinohydrazone (hydramethylnon) as candidate insecticides for post-establishment control of *S. invicta* (Sakamoto and Goka 2021). The results showed that the acute toxicity of fipronil was one to two orders of magnitude stronger than that of the other tested insecticides. Moreover, the results of Argentine ant eradication in Japan (Inoue et al. 2015; Sakamoto et al. 2017, 2019) indicate that it could be effectively used for post-establishment control of *S. invicta.* Nevertheless, fipronil is highly toxic to both invertebrates and vertebrates, so in some environments, insecticides that have less impact may be required.

Considering the ecological characteristics of ants, it is possible to eradicate colonies by terminating the production of the next generation by the queen. Insect growth regulators (IGRs) are control agents that inhibit insect metamorphosis. Because molting failure normally results in insect death, IGRs are typically used as insecticides. The effects of IGRs in ants include a gradual decline in egg production, increased larval and pupal mortality, decreased number of worker ants due to attrition and lack of newly emerged worker ants, abnormal ovaries of queens, and caste shifts. (Glancey et al. 1990; Reimer et al. 1991; Tay and Lee 2014; Vail and Williams 1995). Therefore, IGRs have been used as toxic baits to control *S. invicta* worldwide (Hoffmann et al. 2016; Wylie et al. 2020). However, since IGR agents do not exhibit toxicity to workers, it is not possible to quantitatively evaluate their efficiency in acute toxicity tests. In *S. invicta*, large rearing colonies (including a queen, 40,000–60,000 workers, and 10–40 ml immatures) were used to measure the efficiency of pyriproxyfen (Banks and Lofgren 1991). The colony size was observed to have declined, and the colony died within 6–7 months in the laboratory. It is difficult to directly observe the effect of chemicals on ant larvae when large rearing colonies are used for comparisons of efficiency. Therefore, we constructed a more accurate long-term toxicity assay using artificial colonies to compare the effects of IGRs on the ant larvae under laboratory conditions as other species of ants (Bakr et al. 2005; Vail and Williams 1995; Cabral et al. 2017).

In Japan, the rearing and transport of *S. invicta* are legally prohibited (Ministry of the Environment 2005). Furthermore, *S. invicta* can be harmful to humans, making it difficult to obtain permission to rear this species in Japan. Also, because of the COVID-19 global pandemic, international travel restrictions have made it difficult to enter other countries, including those invaded by *S. invicta*, to conduct the relevant experiments.

Therefore, we attempted to establish a colony-level assay system by using as a substitute species *Tetramorium tsushimae* Emery (Hymenoptera: Formicidae), a Japanese native ant species belonging to the same subfamily (Myrmicinae) as *S. invicta*. Similar to *S. invicta*, *T. tsushimae* is an omnivorous species that prefers both plant and animal materials and has characteristics that make it a potentially invasive alien ant, including having both monogyne and polygyne within the species (Japanese Ant Database Group 2003; Steiner et al., 2006). *Tetramorium tsushimae* is native to Asia but has become an invasive alien ant species in North America (Steiner et al. 2006) . The workers of *T. tsushimae* are approximately 2.5 mm long—about the same size as small workers of *S. invicta*. First, we conducted an acute toxicity assay with fipronil in the same way as for *S. invicta* (Sakamoto and Goka 2021), and we confirmed that *T. tsushimae* was as tolerant as, or slightly more tolerant than, *S. invicta* to fipronil. Then, in this study, we compared the long-term toxicity of insecticides added to an artificial diet by using artificial colonies of 100 workers and 100 larvae of *T. tsushimae*. We examined fipronil and two IGRs: pyriproxyfen, which has been used to control *S. invicta* worldwide, and etoxazole, which has been shown to be effective in colony tests on the large earth bumblebee *Bombus terrestris* (Linnaeus) (Hymenoptera: Apidae) (Besard et al. 2010).

## Materials and methods

### Ants

Three colonies of *T. tsushimae* were collected in Tsukuba City, Japan (140° 04′ 35′′ N, 36° 05′ 31′′E) and held in plastic boxes (19× 14 × 6 cm) until the toxicity test. Each box held a 15-mL distillation centrifuge tube filled with tap water and plugged with absorbent cotton as a water supply, and an insect-pet food jelly (Fujikon, Osaka, Japan) as a food resource. The food jelly was changed twice a week. The ants were held in a rearing room before and during the experiments (25 °C; 70% RH).

### Chemicals

The experimental treatments included fipronil and two IGRs (pyriproxyfen and etoxazole). Fipronil and etoxazole were purchased from Kanto Chemical (Tokyo, Japan) and pyriproxyfen was purchased from Sumitomo Chemical (Tokyo, Japan). To prepare the toxic baits, sugars (powdered sugar, condensed milk), proteins (fish based and insect based), CMC as a base, a small amount of potassium sorbate, and water were mixed and shaped by extrusion granulation. The ratios for each component are listed in Table 1. The product was soaked with an insect control agent at specified doses dissolved in acetone (5% w/w), rice bran oil (0.7% w/w) and antioxidant (0.01% w/w). The solvent was then removed by drying. Cornstarch (1.5% w/w) and powdered sugars (0.8% w/w) were then added to the surface. The baits formed using these materials were observed to be preferred by *S. invicta* (Y. Baba, personal observation). In accordance with the formulation of commercial toxic baits for ants, the concentration of each insecticide in the bait agent was adjusted to 0.05% for fipronil and 0.5% for IGRs.

**Table 1:** Ratio of toxic-bait components used in the experiment

Components	% (w/w)
Proteins (fish based and insect based)	40.0
Sugars (powdered sugar and condensed milk)	50.1
Substrate [carboxymethyl cellulose (CMC) and corn starch]	9.7
Preservative (solbic acid)	0.2

### Acute toxicity assay

Acute toxicity assays to determine the sensitivity of *T. tsushimae* to fipronil were conducted according to Sakamoto and Goka (2021). Fipronil was delivered in 1 µL of acetone (10, 1.0, 0.5 and 0.1 ppm) to the dorsal thorax of each ant, which was anesthetized using ice. Ten ants were examined at each concentration. The treated ants were then dropped individually into plastic cups with moist filter paper and reared for 72 h to determine mortality rates. On the basis of the mortality rates for each concentration of fipronil, the lethal dose for 50% of the population (LD_50_) was calculated every 24 h until 72 h using probit analysis in the R (ver. 3.4.2.) software package.

### Colony-level long-term toxicity assay

We filled the bottom of a polystyrene container (100 × 65 × 28 mm) (As One, Osaka, Japan) with plaster to a thickness of about 10 mm as an artificial nest for our colony-level assays. The plaster was moistened with pure water and then covered with a lid to maintain humidity. We also prepared a polystyrene container (92 × 53 × 96 mm) (Yamada Chemical, Mie, Japan) as a feeding case for the ants, and the two containers were connected by two 6 mm diameter polyethylene tubes to allow the ants to enter and exit. We applied insect barrier paint (Fluon^®^, AGC Chemicals, Tokyo, Japan) to the upper 80 mm of the feeding case, so the ants could not escape. Just before the experiment, each colony of *T. tsushimae* was anesthetized by placing its rearing box in a large polystyrene foam box (33 × 24 × 11 cm) filled with crushed ice. One hundred anesthetized workers and 100 anesthetized larvae per artificial colony were transferred to inside each artificial nest. Four artificial colonies were produced from one field-collected colony of *T. tsushimae*, one for each insecticide and the control. These artificial colonies of the same original colony were kept under the same colony numbers. At the same time, a 5 mL distillation centrifuge tube filled with tap water and plugged with absorbent cotton as a water supply and a 2 cm diameter dish filled with 13 mg of bait were placed in the feeding case. The water supply tube and food dish were replaced with new ones every 3 days. At the same time, the number of surviving individuals was counted for each stage (larvae, pre-pupae, pupae, and emerging new workers). The emerging new workers were distinguished by the color of their cuticle (they are light brown; old workers are black). To avoid affecting the assay by increasing the number of workers, the emerging workers were removed immediately after being counted. The artificial colonies were held in a rearing room during the experiments (25 °C; 70% RH). The observation period was set at 60 days. However, for colonies that still had larvae after 60 days, observation was continued until all larvae became adults or the colony collapsed. For each insecticide, observations were made in three replicates.

## Results

### Acute toxicity of fipronil against T. tsushimae

The LD_50_ values of fipronil were of the same order of magnitude between *S. invicta* (the date was taken from Sakamoto and Goka (2021) and *T. tsushimae* at 24, 48, and 72 h (Table 2). However, *S. invicta* had lower LD_50_ values at all times; in other words, *S. invicta* was slightly more sensitive to fipronil than *T. tsushimae*. The LD_50_ values at 24 h were 1.1 ng/individual for *S. invicta* and 5.2 ng/individual for *T. tsushimae*, but after 72 h they were 0.5 ng/individual for *S. invicta* and 0.7 ng/individual for *T. tsushimae*. The cold anesthesia and acetone control had no negative effect (*N* = 10 each).

**Table 2:** LD_50_ values of fipronil against *S. invicta* and *T. tsushimae* in the 24, 48, and 72 h after exposure

Species	LD_50_ ± SE (LD_10_ ± SE, LD_90_ ± SE) (ng/ant)		
	24 h	48 h	72 h
S. invicta*	1.1 ± 56.0 (0.9 ± 69.8, 1.2 ± 223.6)	0.6 ± 0.1 (0.3 ± 0.1, 1.0 ± 0.2)	0.5 ± 0.1 (0.3 ± 0.1, 1.0 ± 0.3)
*T. tsuhimae*	5.2 ± 1.9 (1.3 ± 0.6, 21.5 ± 12.0)	1.3 ± 0.4 (0.4 ± 0.2, 4.0 ± 2.4)	0.7 ± 0.3 (0.1 ± 0.1, 4.8 ± 3.1)

* The dataset of *S. invicta* was taken from Sakamoto and Goka (2021)

### Effect of toxic baits against worker ants of T. tsushimae

**Fig. 1:**
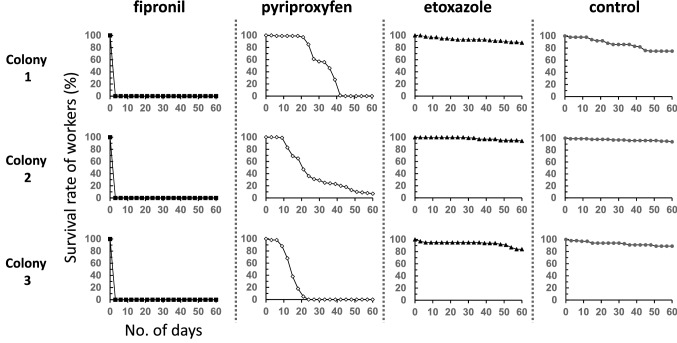
Survival rates of workers in the colony-level assay of *T. tsushimae* (*N* = 3 each). Colonies with the same number indicate colonies separated from the original field colony of the same number

We plotted the survival rates of workers for up to 60 days after the initiation of each insecticide treatment (Fig. 1). In the fipronil-treated colonies (*N* = 3), all workers died within 3 days of treatment initiation. After all workers died, the body color of all remaining larvae changed from white to murky yellow–brown. In the control and etoxazole-treated colonies, more than 80% of the workers in all colonies were still alive after 60 days of observation. In all pyriproxyfen-treated colonies, the survival rate of workers was below 80% at 21 days. Furthermore, after 60 days, two of the three colonies had no workers and had collapsed. In the remaining colony, only two workers survived.

### Effect of toxic baits against emerging new workers

**Fig. 2:**
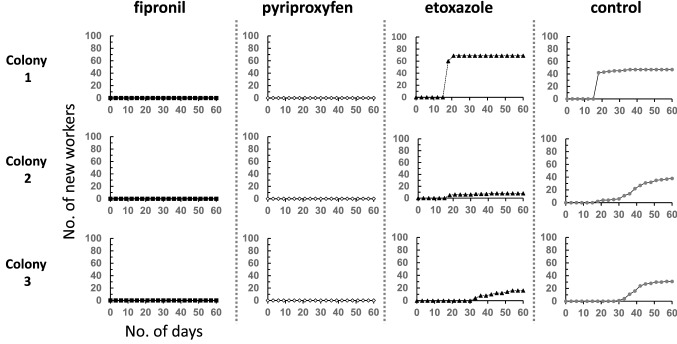
Numbers of new workers in the colony-level assay of *T. tsushimae* (*N* = 3 each). Colonies with the same number indicate colonies separated from the original field colony of the same number

In the etoxazole-treated and control colonies, emerging workers were observed 18 days after the start of the experiment (Fig. 2). In the pyriproxyfen-treated colonies, no new workers had emerged by 60 days. After 104 days of continuous observation of the pyriproxyfen-treated colonies, all workers were dead, and no newly emerged workers had been observed. Also, in the fipronil-treated colonies, all workers died within 3 days of treatment initiation, and all larvae died before pupation.

## Discussion

### Fipronil sensitivity of S. invicta and T. tsushimae

In the acute toxicity assay of fipronil, the LD_50_ values at 24, 48, and 72 h were of the same order of magnitude between *S. invicta* and *T. tsushimae*, but the LD_50_ values were slightly higher in *T. tsushimae* at all times (Table 2). At 24 h, the SE was notably high probably due to a rapid rise in mortality at 10 ppm of fipronil (Fig. S1). On the other hand, the SEs decreased significantly after 48 and 72 h, due to a mild rise in mortality with increasing concentrations of fipronil. Other insecticides need to be examined as well, but, at least for fipronil, *T. tsushimae* should be a suitable substitute for *S. invicta* for use in toxicity assays. Additionally, no negative effects of cold anesthesia were observed in the experimental manipulation using *T. tsushimae* (*N* = 10). In addition, while being reared on an artificial diet for 60 days, which was also preferred by *S*. *invicta* (Y. Baba, personal observation), approximately 80% of workers survived in the control group, indicating that the colony-level assay can be performed stably in polyphagous *T. tsushimae* in laboratory. Furthermore, *T*. *tsushimae* is known to be distributed not only in Japan, but also on the Korean Peninsula and in temperate China (Guénard et al. 2017; Janicki et al. 2016), making it a good substitute model for *S. invicta* for toxicity assays in Asian countries, especially South Korea and Japan, where the number of *S. invicta* invasions has been increasing in recent years and the need for countermeasures is becoming more urgent. (Lyu and Lee 2017; Sakamoto and Goka 2021; Ujiyama and Tsuji 2018). Meanwhile, ecological differences between the two species should be considered when considering the effects of toxic baits at the field level. The numbers of *S. invicta* workers can reach 400,000 in mature colonies (Tschinkel, 2006), whereas that of *T. tsushimae* workers is less than 10,000 (Terayama, 2022). In addition, workers of *T. tsuhimae* are monomorphic with a body length of approximately 2.5 mm (Japanese Ant Database Group 2003), whereas workers of *S. invicta* are continuously polymorphic with a body length of 2.5–6 mm (Tschinkel, 2006). Social complexity based on such worker polymorphisms may affect the results of large-scale colony controls in the field.

### Effectiveness of fipronil for post-establishment control of S. invicta

Even when administered as a toxic bait, fipronil had a strong effect, and all workers of three colonies in the experiment were eliminated within only 3 days (Fig. 1). Additionally, for all colonies, we observed that the body color of the remaining larvae turned from white to murky yellow–brown within one day after all workers died. Generally, in larvae of social hymenopteran insects, the cuticle is not fully sclerotized and melanized, and is highly susceptible to pathogens, and daily grooming by workers is essential for survival (Hölldobler and Wilson 1990). Thus, larvae that changed color were considered to be dead. Acute toxicity assays have already shown that the LD_50_ value of fipronil as a liquid formulation is one to two orders of magnitude smaller than those of other insecticides (Sakamoto and Goka 2021). Therefore, fipronil, which is currently used in Japan as a liquid formulation and toxic-bait agent for control of *S. invicta* (Ministry of the Environment 2021b; Sakamoto et al. 2022), can be considered the most effective insecticide for both liquid and bait control.

### Effectiveness of IGRs for post-establishment control of S. invicta

In all three pyriproxyfen-treated colonies, no new workers were observed (Fig. 2). In contrast, new workers were observed in all control- or etoxazole-treated colonies, beginning at 18 days. No new worker was observed in the pyriproxyfen-treated colonies, even after 102 days. From these results, we concluded that pyriproxyfen is extremely effective as an IGR in inhibiting metamorphosis of *T. tsushimae*. However, the effect of etoxazole varied among the three colonies (Fig. 2). In colony 1, the number of new workers was higher in the etoxazole-treated colonies than in the controls, that is, no IGR effect was observed. In contrast, in colonies 2 and 3, the number of new workers in the etoxazole-treated colonies was approximately half (colony 3) or less than that of the controls (colony 2). In the control, the day of maximum increase in adult numbers differed among colonies (day 18 in colony 1, day 39 in colony 2, and day 42 in colony 3), suggesting that colony 1 had older larvae than colonies 2 and 3. Therefore, etoxazole may exert a growth-regulating effect when ingested long term from young larvae, and further studies are warranted. Nevertheless, in etoxazole-treated colonies 2 and 3, an increase in adults was observed after day 40 (day 42 in colony 2 and day 54 in colony 3), the effect of etoxazole was considered to be weaker than that of pyriproxyfen as IGRs.

**Fig. 3:**
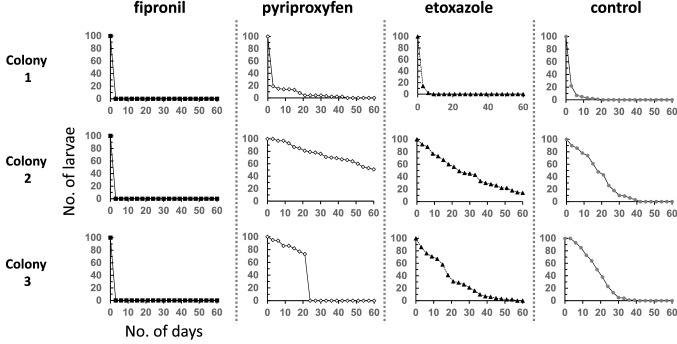
Numbers of larvae in the colony-level assay of *T. tsushimae* (*N* = 3 each). Colonies with the same number indicate colonies separated from the original field colony of the same number.

Negative effects on workers were not observed for etoxazole, but, with pyriproxyfen the number of workers clearly decreased in all treated colonies after 12 days of treatment, and by 60 days only one colony with two workers still survived (Fig. 1). While worker ants remain, the number of larvae in the pyriproxyfen-treated colonies was higher than that in the control and etoxazole-treated colonies from about day 3 to at least day 60 (Fig. 3), indicating that indirect effects caused by a lack of food exchange from the larvae to workers were unlikely to be the causal factor of the decrease in numbers of workers. Chronic toxicity of pyriproxyfen to workers has been reported in the pharaoh ant, *Monomorium pharaonis* (Linnaeus) (Hymenoptera: Formicidae), and in the electric ant, *Wasmannia auropunctata* (Roger) (Hymenoptera: Formicidae) which belong to the subfamily Myrmicinae, the same subfamily to which *T. tsushimae* and *S. invicta* belong (Bakr et al. 2005; Vail and Williams 1995; Cabral et al. 2017). In an artificial colony composed of pharaoh ant workers, toxic bait with 0.1% pyriproxyfen killed all workers within 16–22 days (Bakr et al. 2005; Vail and Williams 1995). In contrast, in the electric ant, worker numbers were not significantly different from the control after six weeks of feeding toxic baits with 0.5% pyriproxyfen (Cabral et al. 2017). In social hymenopteran insects, the function of juvenile hormones in adults remains unclear, but they have been shown to be involved in reproductive and behavioral control (Robinson and Vargo 1997; Shpigler et al. 2020). Therefore, the administration of pyriproxyfen, a juvenile hormone analog, may have disrupted juvenile hormone regulation in the workers, resulting in chronic toxicity. In contrast, etoxazole, which inhibits molting by inhibiting chitin synthesis (Nauen and Smagghe 2006), was not expected to have such chronic toxicity, even though it is also classified as an IGR. It has been previously reported that *S. invicta* will move its nests if the number of workers is reduced by the effects of insecticides (Collins and Callcott 1995). In other words, negative effects on workers may encourage the migration of *S. invicta* that detect abnormalities in the colonies, so continuous monitoring of the distribution dynamics of the colony is necessary.

### Post-establishment control of S. invicta using fipronil and pyriproxyfen

From investigations of *S. invicta* colony control cases in New Zealand (Sarty 2007) and Australia (Hoffmann et al. 2016), as well as cases of successful eradication of Argentine ants in Japan (Inoue et al. 2015; Sakamoto et al. 2017, 2019), we believe that the combined use of a liquid formulation treatment and baiting is effective for controlling invasive alien ants. In post-establishment control, it is necessary to assume two stages: emergency control when the initial nests are found (e.g., in ports in the early stages of invasion) and long-term control after several mature nests have been found.

**Fig. 4:**
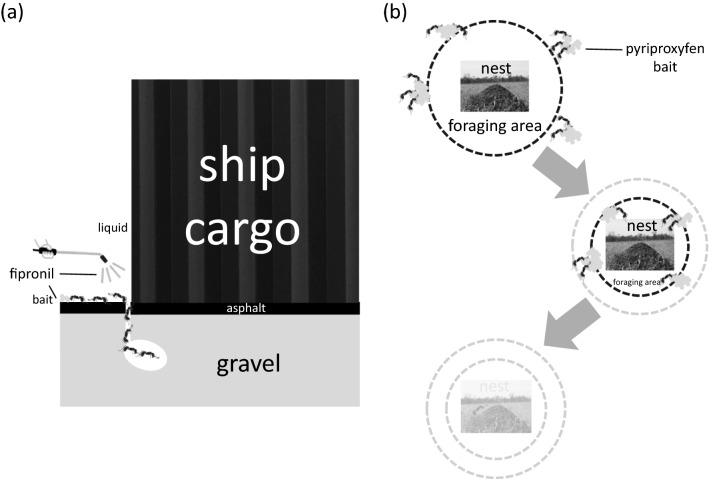
Schematic diagram of post-establishment control of *S. invicta*: **a** emergency control and **b** long-term control

Fipronil, which is the most promising liquid formulation candidate for emergency control in port areas, is considered to be highly toxic to *S. invicta*, via contact and when ingested (Sakamoto and Goka 2021, this study). Therefore, when a wild nest of *S. invicta* is found, it is advisable to inject fipronil into the nest as a method of emergency control. Because fipronil has a high residual effect, it is expected to have a sustained effect in controlling reinvasion by *S. invicta* from overseas. However, areas that the liquid formulation alone cannot reach may remain in the nest, or some individuals may escape and disperse. Therefore, toxic baits should also be placed around the nest to continuously stop the production of queens and larvae in the nest (Fig. 4a). Our study showed that pyriproxyfen is effective for such long-term suppression. Pyriproxyfen baits have been successfully used in colony-level control against *S. invicta* in formerly invaded countries (Hwang, 2009; Sarty, 2007; Vanderwoude et al., 2003; Williams et al. 2001). However, colony disruption by pyriproxyfen may take several weeks (Fig. 1), so where there is concern about *S. invicta* stinging people, fipronil baits should be used to control *S. invicta* to reduce the number of workers rapidly and thus minimize human injury.

For future long-term control, when *S. invicta* are building mature nests, chemical control must be implemented, with the primary goal of avoiding the spread of *S. invicta* through unnecessary stimulation of its nests. In this case, as has been done with Argentine ants (Inoue et al. 2015; Sakamoto et al. 2017, 2019), the foraging range should be monitored and toxic baits placed at its border to gradually reduce the size of the colony (Fig. 4b). Pyriproxyfen has a less negative impact on vertebrates (Pener and Dhadialla 2012) and would be appropriate as a bait for widespread application. Rapid implementation of chemical control by using these effective agents is desirable. The acute toxicity assay and colony-level toxicity assay methods we have improved (Sakamoto and Goka 2021; this study) will allow us to select more effective insecticides and develop new insecticides for ants in the future. Incorporating such agents should contribute to improved chemical control of the establishment of *S. invicta*.

## Supplementary Information

Below is the link to the electronic supplementary material.

Supplementary file1 (PPTX 75 KB)
