# Supplementary material for: Efficiency of ant-control agents in colony-level oral toxicity tests using Tetramorium tsushimae (Hymenoptera: Formicidae) for post-establishment control of the red imported fire ant, Solenopsis invicta (Hymenoptera: Formicidae)
Source: Appl Entomol Zool. 2022 Oct 9;58(1):25–33. doi: 10.1007/s13355-022-00800-x (PMC9547751; doi:10.1007/s13355-022-00800-x)
Supplement: Supplementary file 1 — Supplementary file1 (PPTX 75 KB) [file 13355_2022_800_MOESM1_ESM.pptx]

## Slide 1
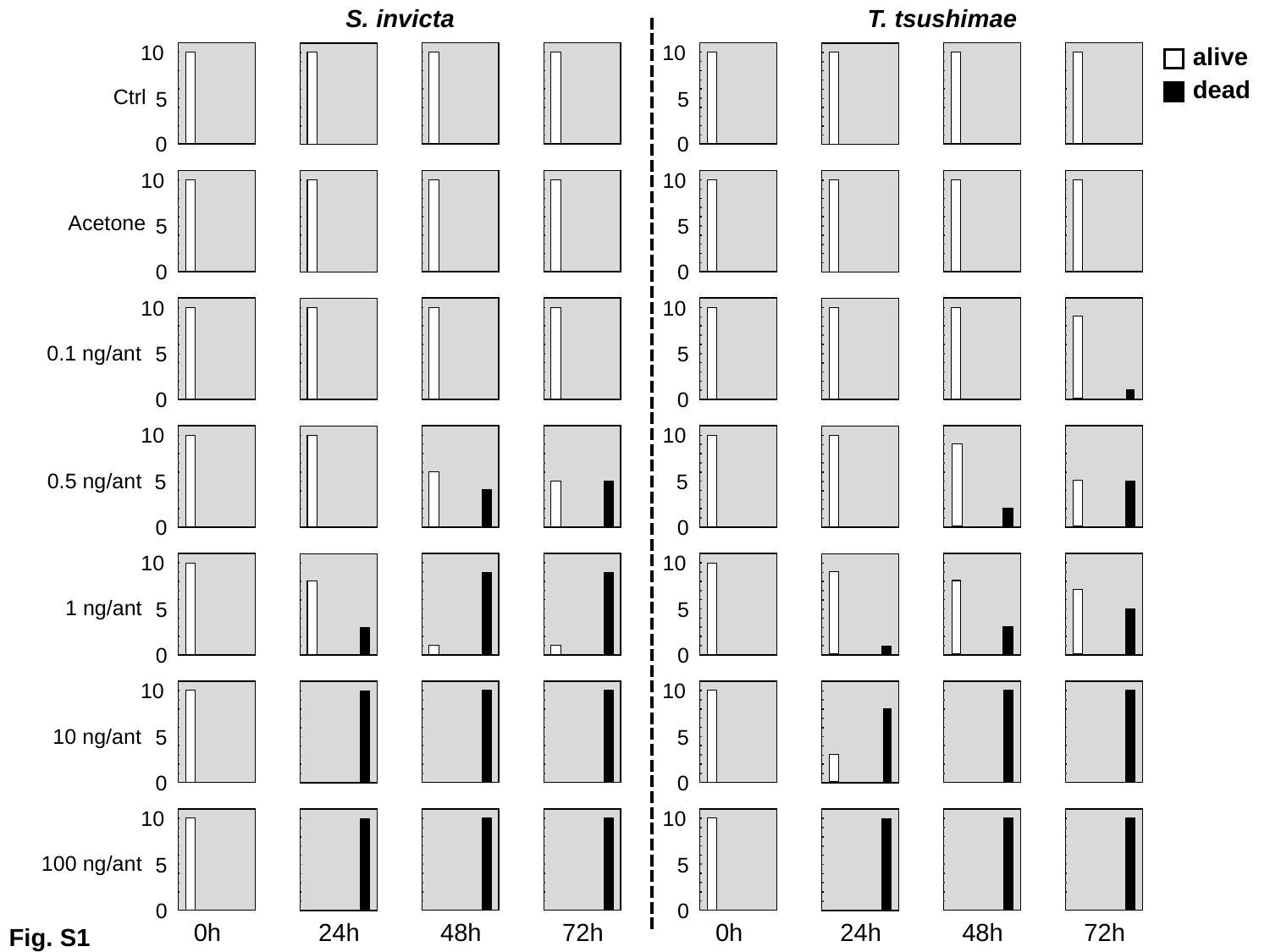

S. invicta
10
5
0
10
5
0
10
5
0
10
5
0
10
5
0
10
5
0
10
0h
5
0
48h
72h
24h
T. tsushimae
10
5
0
10
5
0
10
5
0
10
5
0
10
5
0
10
5
0
10
0h
5
0
48h
72h
24h
alive
dead
Ctrl
Acetone
0.1 ng/ant
0.5 ng/ant
1 ng/ant
10 ng/ant
100 ng/ant
Fig. S1
